# Extratesticular masses focusing on MRI findings

**DOI:** 10.1007/s11604-024-01605-4

**Published:** 2024-06-05

**Authors:** Hiromi Edo, Fumiko Yagi, Mariko Mizuno, Masahiro Okada, Eiko Hyoe, Ippei Ozaki, Hirotaka Akita, Masahiro Jinzaki, Hiroshi Shinmoto

**Affiliations:** 1 Department of Radiology, National Defense Medical College, 3-2 Namiki, Tokorozawa-shi, Saitama 359-8513 Japan; 2 Department of Diagnostic Radiology, Keio University School of Medicine, 35 Shinanomachi, Shinjuku-ku, Tokyo 160-8582 Japan; 3 Department of Radiology, Nihon University School of Medicine, 30-1 Oyaguchikami-Machi, Itabashi-ku, Tokyo 173-8610 Japan

**Keywords:** Extratesticular mass, Paratesticular mass, MRI, Scrotum, Spermatic cord

## Abstract

Scrotal masses, whether cystic or solid lesions, are routinely evaluated using ultrasonography. Magnetic resonance imaging (MRI) may be used for further investigation in cases with atypical findings, difficult diagnoses, large masses, and/or unclear relationships with the surrounding tissues. Scrotal solid masses are divided into intra- and extra-testicular masses. A staggering 90% of the intratesticular masses are malignant, whereas 75% of extratesticular masses are benign. Extratesticular masses are less common than intratesticular masses; however, some extratesticular masses present characteristic MRI findings. Familiarity with these specific MRI features of extratesticular masses is beneficial to radiologists, as appropriate diagnoses can help avoid unnecessary invasive treatments such as orchiectomy. In this review, we describe fibrous pseudotumors, polyorchidism, adenomatoid tumors, and scrotal leiomyoma as benign paratesticular masses, focusing on their characteristic imaging features on MRI. Although these tumors are extremely rare, their MRI findings are distinctive, and accurate diagnoses can prevent unnecessary orchiectomy. In addition, to demonstrate the pitfalls of diagnosing extratesticular masses, we present a case of seminoma misidentified as extratesticular masses due to large extensions outside the testis. Spermatic cord sarcoma, including rhabdomyosarcoma, leiomyosarcoma, and liposarcoma, and metastasis to the spermatic cord are described as malignant extratesticular masses. This review focused on extratesticular masses and elaborates the imaging findings that can aid in the accurate diagnosis using MRI.

## Introduction

Scrotal masses, whether cystic or solid lesions, are usually evaluated using ultrasonography. If a benign extratesticular cystic mass, such as a spermatocele or an epididymal cyst, is determined through ultrasonography, further evaluation with magnetic resonance imaging (MRI) is not necessary. MRI may be performed for further examination in cases of atypical findings, difficult diagnoses, large masses, or unclear relationships with adjacent tissues. The most important step in diagnosing an intra-scrotal solid mass is to determine its location, whether it is an intra- or extra-testicular mass. This is because 90% of intratesticular tumors are malignant, whereas 75% of extratesticular masses are benign, and the differential diagnosis are completely different [1, 2]. In addition, some extratesticular masses, although rare, have characteristic MRI findings. MRI can play a major role in accurate diagnosis and can prevent unnecessary orchiectomy, which is clinically significant. This review summarizes MRI findings of extratesticular solid masses in the scrotum that are clinically useful to radiologists.

## Normal anatomy of the scrotum on MRI

The scrotum, an external pouch of skin and muscles located below the pelvis, houses the testicles, epididymis, and part of the spermatic cord. The testicles are responsible for sperm production and hormone secretion, while the epididymis facilitates sperm maturation and storage. The spermatic cord contains various structures, including the vas deferens, blood vessels, nerves, and lymphatic vessels, sustaining the testicles. MRI can effectively visualize these anatomical structures, providing detailed information about their morphology and detecting any abnormalities.

**Fig. 1:**
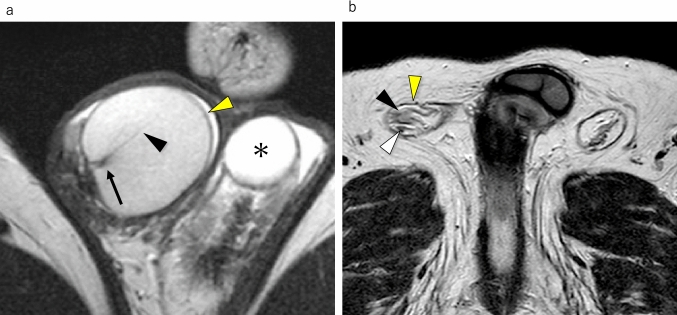
Normal anatomy of the scrotum on MRI. **a** Axial T2-weighted MR image shows a right testis enveloping tunica albuginea (yellow arrowhead) recognizing as a low-signal structure. Within the testis, the septa (black arrowhead) separating the lobules are observed as linear low-signal structure, continuous to a low-signal structure called the testicular mediastinum (black arrow). In the left scrotum, a spermatocele (asterisk) is observed. **b** Axial T2-weighted MR image shows the spermatic cords. The spermatic cord is a cord-like structure that contains nerves, blood vessels (black arrowhead), and the vas deferens (white arrowhead). The spermatic cord is enveloped by a layer (yellow arrowhead) comprising the cremasteric muscle sandwiched between internal and external spermatic fasciae

The testes are enveloped by the tunica albuginea and the testicular parenchyma is divided by septa. The mediastinum of the testes lies at the center of these septa, from which approximately 20 ducts extend and connect to the epididymis. The epididymis comprises the head, body, and tail, with the tail continuing into the vas deferens. The testicular artery and vein also enter the testes near the mediastinum. This region is referred to as the testicular hilum. Portions of the tunica albuginea, septa, and mediastinum of the testis are identified on MR image using T2WI (Fig. 1a).

The spermatic cord constitutes one of the components of the scrotum. It is a cylindrical structure containing nerves, blood vessels, and the vas deferens. The spermatic cord is encased by a complex layer comprising the cremasteric muscle ensconced between the internal and external spermatic fasciae. The anatomical components comprising the spermatic cord are identified on T2WI (Fig. 1b).

## Descent of the testis and epididymis, and development of the tunica vaginalis

**Fig. 2:**
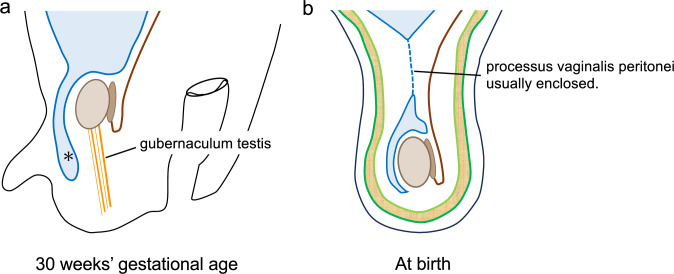
Descent of the testis and epididymis, and development of the tunica vaginalis. **a** At 30 weeks of gestation, the testicle is pulled by the gubernaculum testis, descending into the scrotum; concurrently, the peritoneum protrudes into the scrotum as the processus vaginalis peritonei (*). **b** At birth, processus vaginalis peritonei is usually enclosed (dotted line)

The descending testis and development of the tunica vaginalis are depicted in Fig. 2. The “testis” and the “mesonephric duct, which later becomes the epididymis,” are initially attached to the posterior abdominal wall. At approximately 30 weeks of gestation, the testis and epididymis are guided by the gubernaculum testis and migrate into the scrotum. The peritoneum extends into the inguinal canal along with the descending testes to form the processus vaginalis peritonei. At birth, the processus vaginalis peritonei is closed, except for the tunica vaginalis. Therefore, the tunica vaginalis is a remnant of the original peritoneum. The hydrocele of the testis is a fluid collection in the area surrounded by the tunica vaginalis.

## Anatomy of the scrotum and the spermatic cord, focusing on the fascia

**Fig. 3:**
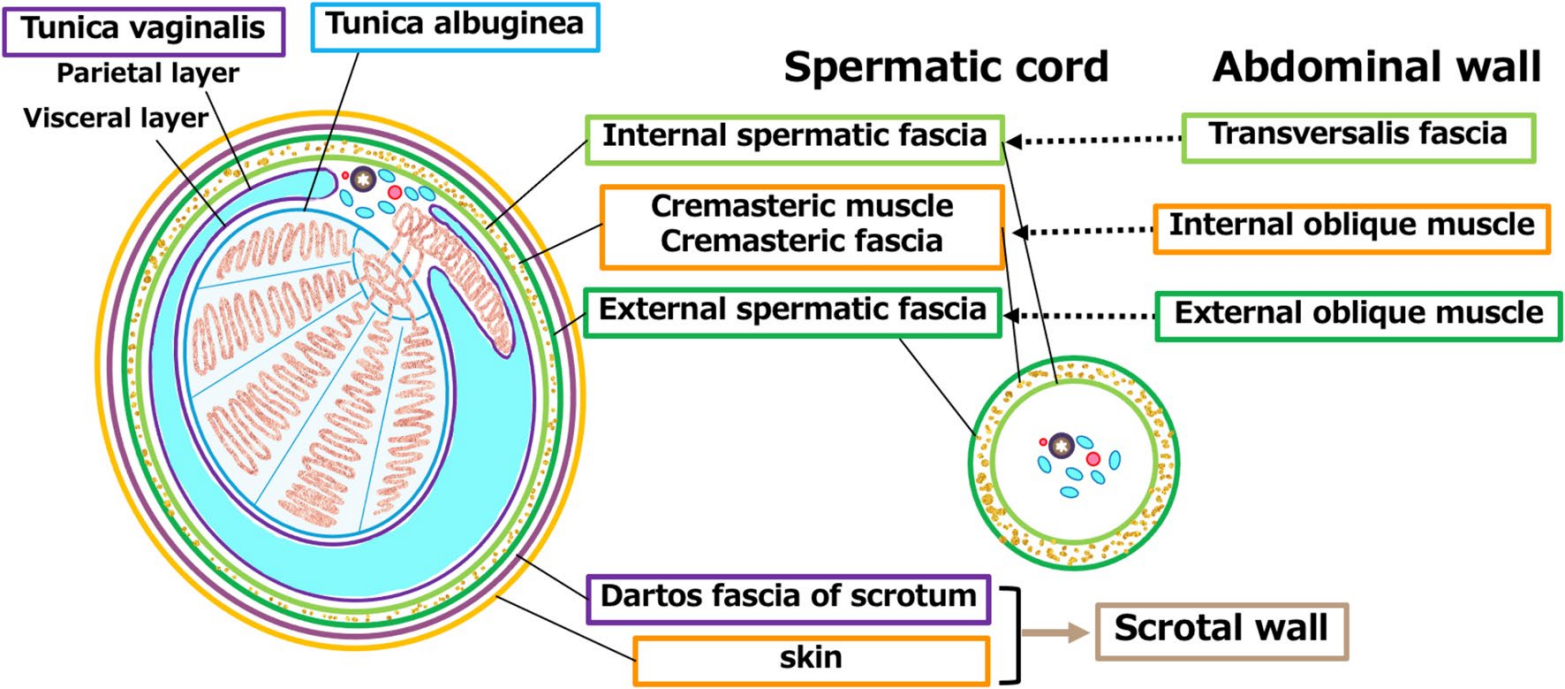
Anatomy of the scrotum and the spermatic cord, focusing on fascia. The testicular parenchyma is covered by the tunica albuginea, while the tunica vaginalis, a remnant of the peritoneum, covers the surfaces of the testis and epididymis. These structures are surrounded by the cremasteric muscle, which is sandwiched between the internal spermatic fascia and the external spermatic fascia. The spermatic cord contains the testicular artery, vein, nerves, and vas deferens, surrounded by cremasteric muscles and layers of spermatic fascia. These fascial structures are extensions of the abdominal wall muscles, with the transversalis fascia forming the internal spermatic fascia, the internal oblique muscle forming the cremasteric muscle, and the external oblique muscle forming the external spermatic fascia. The outermost layer includes the Dartos fascia and skin, forming the scrotal wall

An anatomical schema focusing on the fascia of the scrotum and spermatic cord is shown in Fig. 3. The tunica albuginea envelopes the testicular parenchyma, whereas the tunica vaginalis, the remnant of the peritoneum, covers the surfaces of the testis and epididymis. The tunica albuginea and tunica vaginalis are essentially located around the periphery of the testis. The surrounding area includes various membranes that add complexity to the structure. The spermatic cord comprises the cremasteric muscle surrounded by the spermatic fascia both internally and externally, resulting in a layered structure that encloses the testicular vessels. Additionally, on the outermost layer, the Dartos fascia (subcutaneous tissue) and skin form the scrotal wall. The spermatic cord is composed of the testicular artery, vein, nerves, and vas deferens, and is surrounded by cremasteric muscles and the internal and external layers of the spermatic fascia. The duct leaves the epididymis, along with the testicular artery and vein, and moves towards the abdominal wall enveloped by the cremasteric muscle and the internal and external layers of the spermatic fascia; this tubular structure forms the spermatic cord. These fascial structures enveloping the spermatic cord are extensions of the abdominal wall muscles. The transversalis fascia extends to form the internal spermatic fascia, the internal oblique muscle extends to form the cremasteric muscle, and the external oblique muscle extends to form the external spermatic fascia.

## Key MRI findings in differentiating scrotal masses and pitfalls

The first step is to observe the images to determine whether the lesion is an intra- or extratesticular mass in the scrotum. If it is an intratesticular lesion, since the probability of malignancy is 90%, malignant primary testicular tumors (seminoma or mixed germ cell tumor) and malignant lymphoma are considered first. In case of extratesticular lesions, 75% being benign, unnecessary radical orchiectomy can be avoided if the mass shows the characteristic MRI findings.

**Fig. 4:**
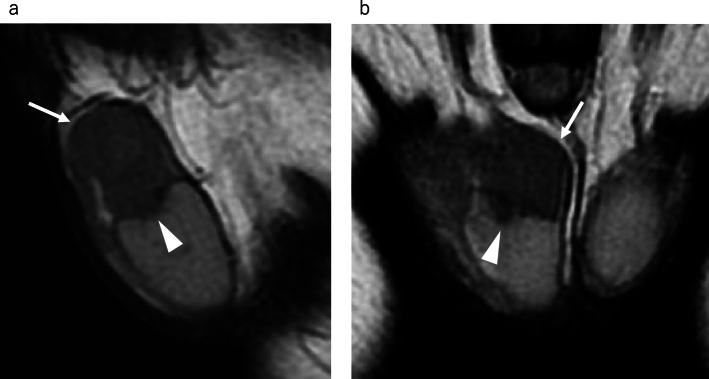
Seminoma in a 43-year-old male, extending into the extratesticular area and mimicking a benign extratesticular mass owing to minimal intratesticular component. **a** Sagittal T2-weighted MR image shows a homogeneous mass above the testis, exhibiting a mild hypointensity compared to the testis, mimicking extratesticular mass (white arrow). Mass is extending to the mediastinum of testis (white arrowhead). The figure is adapted from a study by Edo et al. [34]. **b** Coronal T2-weighted MR image shows a mass (white arrow) extending to the mediastinum of the testis (white arrowhead)

If the mass involves both the intra- and extra- testicular portions, even if the size of the extratesticular portion is large, the possibility of extratesticular extension of a malignant intratesticular tumor, such as a seminoma or non-seminomatous mixed germ cell tumor, should be considered. On MRI, seminoma can be misdiagnosed as a benign extratesticular mass because the lesion is relatively homogeneous and well-defined (Fig. 4). Even if the size of the lesion within the testis is smaller compared to lesions outside the testis, it is necessary to consider the possibility of a malignant testicular tumor.

## Benign paratesticular tumor

### Fibrous pseudotumor

Fibrous pseudotumor is a benign tumor resulting from fibroinflammatory reaction [3, 4]. Although it often originates from the tunica vaginalis, it can also arise from the epididymis or spermatic cord. Fibrous pseudotumor is suggested to be associated with preceding inflammation, with 50% showing a correlation with hydrocele and 30% with a history of trauma or prior epididymo-orchitis [3]. Fibrous pseudotumor may detach, allowing them to move freely, ultimately forming a "scrotal pearl" [3]. Recently, this non-neoplastic disease has been reported to be associated with IgG4-related diseases [5–7]. Given its rarity and potential for clinical misinterpretation as a malignant tumor, preoperative diagnosis is crucial [8]. While it predominantly affects individuals in their 30 s, it can occur across all age groups, with reported sizes ranging from 0.5 to 8 cm, and may present as a solitary or multiple lesions.

**Fig. 5:**
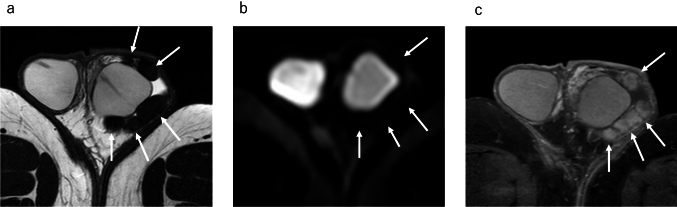
Fibrous pseudotumor in a 28-year-old male with left paratesticular masses. **a** Axial T2-weighted MR image shows marked hypointense multiple masses (white arrows), which are in contact with tunica vaginalis. **b** Axial diffusion weighted MR image shows masses (white arrows) with marked low-signal intensity. It is difficult to identify the location of masses without referring to other sequencing images. **c** Axial contrast-enhanced fat-suppressed T1-weighted MR image shows heterogeneous enhancement of the masses (white arrows)

**Fig. 6:**
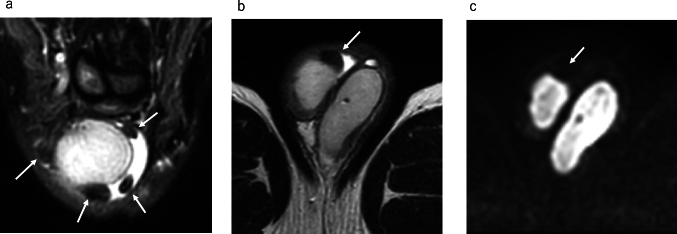
Fibrous pseudotumor in a 53-year-old male with right paratesticular masses accompanied with mild hydrocele. **a** Coronal fat-suppressed T2-weighted image demonstrates hypointense masses (white arrows). **b** Axial T2-weighted image shows a hypointense mass attaching to the tunica vaginalis (white arrow). **c** Axial diffusion weighted MR image shows a hypointense mass (white arrow), and it is difficult to identify the location of mass without referring to other sequencing images

On MRI, the tumor appears as multiple masses surrounding the testis, called paratesticular masses, reflecting its origin from tunica vaginalis. These multiple paratesticular nodules are depicted as very low-signal intensity masses on T2WI, with low-signal intensity on diffusion weighted imaging (DWI); these findings are very specific MRI features (Figs. 5, 6) [3, 4, 9]. Contrast enhancement varies from poor to well-enhanced [3, 4]. Multiple T2WI and DWI low-signal masses in contact with the tunica vaginalis around the testis should be considered as fibrous pseudotumor.

The treatment is tumor excision, and orchiectomy is not necessary [9]. If a fibrous pseudotumor can be diagnosed preoperatively, unnecessary orchiectomy can be avoided and MRI can be very useful.

### Adenomatoid tumor

Adenomatoid tumors are rare, benign lesions of mesothelial origin that account for approximately 30% of all paratesticular masses [10]. They are more frequently observed in individuals from 20 to 50 years [11]. In males, they typically arise from the epididymis (majority), tunica albuginea (14%), or spermatic cord (rare); whereas in females, they originate from the uterus or fallopian tubes. Various theories exist regarding its origin; however, based on immunostaining results, it is derived from mesothelial cells that originate from coelomic epithelial cells [12]. Many cases are asymptomatic, incidentally discovered, and usually observed as a single mass. The tumors are often small, with the majority measuring less than 2 cm, typically ranging from 0.4 to 5 cm [2].

**Fig. 7:**
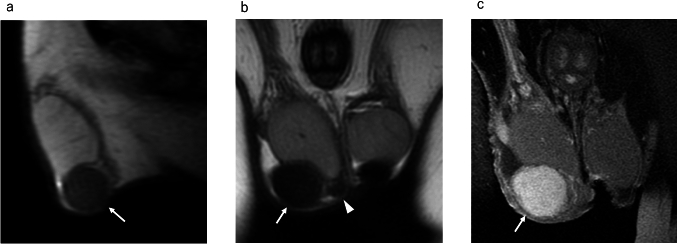
Adenomatoid tumor in a 43-year-old male. **a** Sagittal T2-weighted MR image shows a hypointense mass (white arrow) measuring 2.5 cm. The testis is compressed by the mass without invading the testis parenchyma, indicating that it is an extratesticular mass. **b** Coronal T2-weighted MR image shows a hypointense mass (white arrow) which is suspected to be continuous with the tail of the epididymis (white arrowhead). **c** Coronal contrast-enhanced fat-suppressed T1-weighted MR image shows homogeneous enhancement of the mass (white arrow)

On MRI, typical findings of adenomatoid tumor is localized near the lower pole of the testis and shows a lens-shaped appearance with low signal intensity rim on T2WI [13, 14]. Adenomatoid tumors are located near the lower pole of the testis, possibly because they occur in the tail of the epididymis (Fig. 7a, b). When a lens-shaped appearance occurs at the lower pole of the testis, an adenomatoid tumor can be suspected based on its characteristic MRI findings; however, it can also be circular, oval, or occasionally predominantly cystic [2]. Contrast-enhanced MRI of adenomatoid tumors is characterized by the rim enhancement [13]; however, the entire mass may show a strong contrast-effect (Fig. 7c), and the degree of contrast effect may vary.

Surgical excision is the treatment of choice for adenomatoid tumors. The surgical approach aims to remove the tumor while preserving as much normal testicular tissue as possible. This approach is facilitated by adequate ultrasonography and intraoperative frozen sections [10]. Unnecessary orchiectomy can be avoided if a preoperative diagnosis can be made based on these characteristic MRI findings of adenomatoid tumors.

### Polyorchidism

**Fig. 8:**
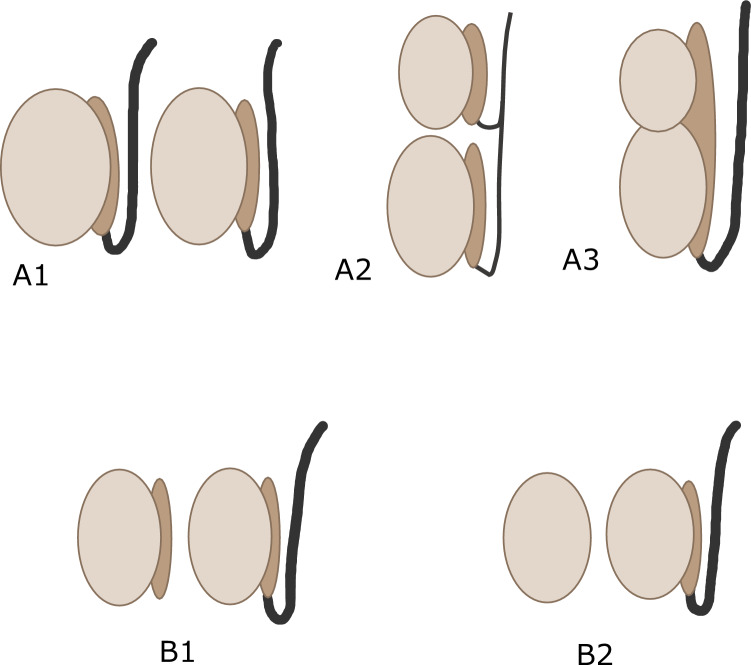
Schema of classification of polyorchidism. **A1** The supernumerary testicle has its own vas deferens and epididymis, making it completely independent of the normal testicle. **A2** The supernumerary testicle shares the vas deferens with the normal testicle but has its own separate epididymis. **A3** The supernumerary testicle shares the vas deferens and epididymis with the normal testicle. **B1** The supernumerary testicle lacks a vas deferens but has its own separate epididymis. **B2** The supernumerary testicle lacks both a vas deferens and an epididymis

Polyorchidism is a rare congenital anomaly characterized by supernumerary testes. Supernumerary testes are most often located in the scrotum (75%) but may also be found in the inguinal canal, retroperitoneum, or intraperitoneal cavity [15, 16]. Furthermore, polyorchidism may impact male fertility due to potential abnormalities in spermatogenesis and sperm quality associated with the additional testes [16]. Polyorchidism is usually classified as type A or B based on the presence or absence and continuity of the epididymis and vas deferens (Fig. 8) [15]. A3 is the most common type.

**Fig. 9:**
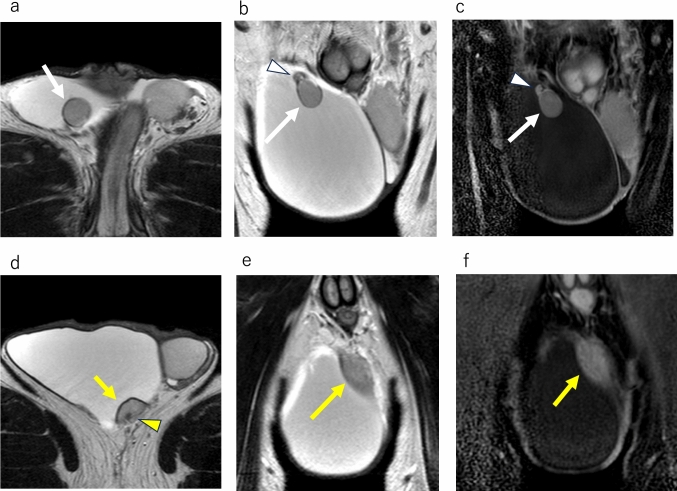
Polyorchidism in a 65-year-old male with supernumerary testis in right scrotum with hydrocele. **a** Axial T2-weighted MR image shows mild atrophied right testis (white arrow). **b** Coronal T2-weighted MR image shows the right testis (white arrow), attached epididymis (white arrowhead) with hydrocele. **c** Coronal contrast-enhanced fat-suppressed T1-weighted MR image shows enhancement of the epididymis (white arrowhead) and right testis (white arrow). **d** Axial T2-weighted MR image shows a mass with equal intensity to the left normal testis. Linear hypointense structure (yellow arrowhead) is observed in the mass. The mass is a supernumerary testis and the internal linear hypointense structure (yellow arrowhead) is the testicular mediastinum. **e** Coronal T2-weighted MR image shows a supernumerary testis (yellow arrow) with hydrocele. **f** Coronal contrast-enhanced fat-suppressed T1-weighted MR image shows enhancement of a supernumerary testis (yellow arrow)

On MRI, the supernumerary testes demonstrate signal intensity and contrast enhancement similar to those of normal testes (Fig. 9). In addition, except for type B2, supernumerary testes have an epididymis and/or vas deferens. The presence of the testicular mediastinum suggests that it may be a supernumerary testis.

There is no established indication for surgery for polyorchidism; however, a supernumerary testis outside the scrotum has a high risk of malignancy and requires excision. Notably, if it is located within the scrotum and there is no torsion or malignant findings, it can be treated conservatively [15, 16]. Since the MRI findings of polyorchidism are quite specific, MRI can help in preoperative diagnosis and avoid unnecessary surgery.

### Scrotal leiomyoma

Scrotal leiomyoma is a rare benign tumor of smooth muscle cells that originates from the Dartos fascia of the scrotum, epididymis, spermatic cord, or tunica albuginea [17]. Although leiomyomas are known to arise from the uterus, scrotal leiomyomas are very rare in men. Scrotal leiomyomas are usually painless slow-growing masses [18]. It can occur at any age but is most likely to occur in individuals in their 40 s [2]. On physical examination, leiomyomas are firm or hard rubbery masses that often adhere to the skin [4]. Scrotal leiomyomas tend to be located in the inguinal canal of the spermatic cord, whereas leiomyosarcomas are often located on the scrotal side of the spermatic cord [3].

**Fig. 10:**
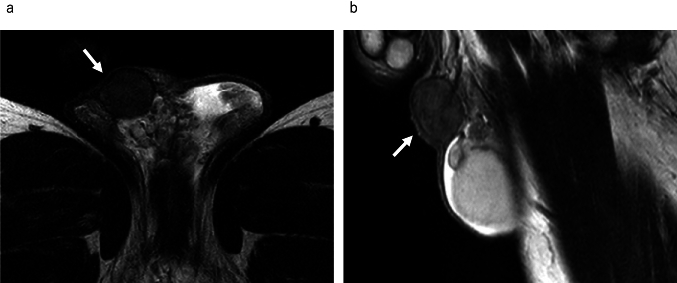
Scrotal leiomyoma in a 40-year-old-male with a scrotal mass recognizing for more than 10 years. **a** Axial T2-weighted MR image shows a homogeneous hypointense mass (white arrow) attached to the Dartos fascia in the right scrotum near the right spermatic cord. **b** Sagittal T2-weighted MR image shows a hypointense mass (white arrow), which is located in the right scrotum above the right testis

On MRI, scrotal leiomyomas, like leiomyomas arising in the uterus, show low signal intensity on T2WI, reflecting smooth muscle [18, 19]. In the case of Dartos fascia origin, the mass is observed to be in contact with the Dartos fascia on MRI (Fig. 10). MRI allows the relationship between the mass and surrounding tissues to be easily understood. The contrast enhanced effect of the mass is variable [18, 19].

The primary treatment is surgical excision of the local lesion [20]. Complete surgical resection of the tumor is curative and recurrence after adequate resection is rare. The surgical approach depends on the size and location of the tumor, but generally involves local excision with preservation of the surrounding structures to minimize both cosmetic and functional after-effects.

## Malignant paratesticular tumor

### Spermatic cord sarcoma

Excluding lipomas, 56% of the spermatic cord masses are malignant [1]. Among spermatic cord sarcomas, Rhabdomyosarcoma and Liposarcoma are the most prevalent, with rhabdomyosarcoma having a higher incidence [3]. Conversely, leiomyosarcoma is infrequent in the context of spermatic cord sarcomas. Rhabdomyosarcomas are more commonly observed in children, whereas other sarcomas occur in older individuals [3]. A consensus regarding the type of surgery and adjuvant treatment is yet to be determined. The rate of local recurrence in the scrotum and groin after orchidectomy ranges from 25 to 37%, indicating the need for aggressive surgery or adjuvant treatment [21]. Wide radical resection remains the first and perhaps the most important step [22]. Negative surgical margins are important to reduce the recurrence rate, and MRI is a useful examination to assess the extent of sarcoma extension.

### Rhabdomyosarcoma

Spermatic cord rhabdomyosarcomas are the most frequently encountered malignant paratesticular tumors, accounting for approximately 40% of all cases. It commonly occurs in infants and children, with peak incidence during the first decade of life. Spermatic cord rhabdomyosarcomas are characterized by their aggressive nature, with approximately 40% of patients presenting with metastasis at diagnosis [2]. Some cases are painful and can be mistaken clinically for epididymitis [23].

**Fig. 11:**
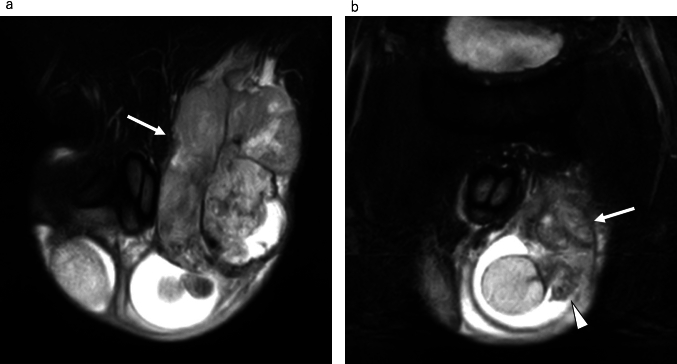
Rhabdomyosarcoma in a 21-year-old male with a left scrotal mass enlargement. **a**, **b** Coronal fat-suppressed T2-weighted MR images shows a heterogeneous hyperintense mass (white arrow) located in the spermatic cord. The mass invades the epididymis (white arrowhead), but the testis is preserved

On MRI, spermatic cord rhabdomyosarcomas may present as heterogeneous masses with variable-signal intensity often with irregular margins and areas of necrosis or hemorrhage (Fig. 11) [24, 25]. However, these imaging features lack specificity and may overlap with those of other extratesticular tumors.

The cornerstone of treatment for paratesticular rhabdomyosarcomas is radical orchiectomy, which involves complete surgical removal of the affected testis and surrounding structures [26]. Cases in which metastases are present or suspected, retroperitoneal lymph node dissection may be indicated to assess and manage regional lymph node involvement. Given the aggressive nature of paratesticular rhabdomyosarcomas, aggressive adjuvant chemotherapy regimens are often employed as part of multimodal therapy to improve outcomes and reduce the risk of recurrence [2, 26].

### Liposarcoma

Liposarcoma of the spermatic cord is a rare malignancy that accounts for approximately 5–7% of spermatic cord sarcomas [27]. Most cases occur in the spermatic cord; however, some arise in the retroperitoneum, extend into the inguinal region, and involve the spermatic cord [28]. Low-grade liposarcomas, including atypical lipomatous tumor (ALT) /well-differentiated liposarcoma, typically spread by local extension and involve adjacent structures within the testis and epididymis. Clinically, low-grade spermatic cord liposarcomas may be mistaken for an inguinal hernia [28]. In contrast, high-grade liposarcomas may exhibit more aggressive behavior and potentially spread via the hematogenous and lymphatic routes to distant sites.

**Fig. 12:**
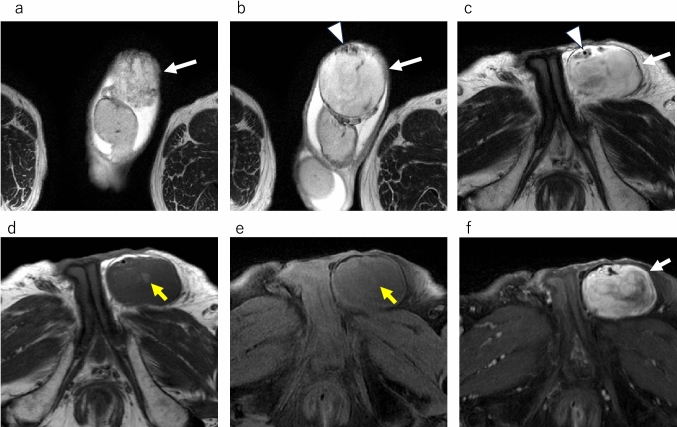
Liposarcoma in an 83-year-old male with the left scrotum swollen. **a**–**c** Axial T2-weighted MR images demonstrate hyperintense mass (white arrow) in the left scrotum. The mass is located in the left inguinal canal, and the mass compresses the spermatic cord (white arrowhead) anteriorly. **d** Axial T1-weighted MR image shows a small hyperintense area (yellow arrow) in the mass. **e** Axial fat-suppressed T1-weighted MR image shows hypointense area (yellow arrow) that is hyperintense on T1-weighted MR image (**d**, yellow arrow), suggesting that the tumor contains fat component. **f** Axial contrast-enhanced fat-suppressed T1-weighted MR image shows heterogeneous enhancement of the mass (white arrow)

MRI findings of spermatic cord liposarcoma vary based on the degree of differentiation. Low-grade liposarcoma has mostly fatty component with focal enhancing components that are suspicious for malignancy. In contrast, a high-grade liposarcoma may be a heterogeneous solid mass with only a small fatty component. In both low- and high-grade liposarcomas, the presence of a fat component on computed tomography (CT) or MRI is the basis for diagnosis. The presence of a fat component within the lesion, as detected by imaging modalities, such as MRI, can aid in distinguishing these tumors from other extratesticular masses (Fig. 12).

The primary treatment for spermatic cord liposarcomas is radical orchiectomy, which involves the complete surgical removal of the affected testis and surrounding structures, to achieve local control. Adjuvant radiation therapy is typically recommended for intermediate- or high-grade lesions to target the residual disease and reduce the risk of local recurrence. In addition to radiation therapy, postoperative adjuvant chemotherapy may be considered, particularly in cases with high-risk features or evidence of systemic spread [2].

### Leiomyosarcoma

Spermatic cord leiomyosarcoma is a rare malignancy that accounts for approximately 10% of all spermatic cord sarcomas. These tumors typically originate in the scrotal part of the spermatic cord. This is in contrast with leiomyomas, which are more commonly located in the inguinal region of the spermatic cord [3].

**Fig. 13:**
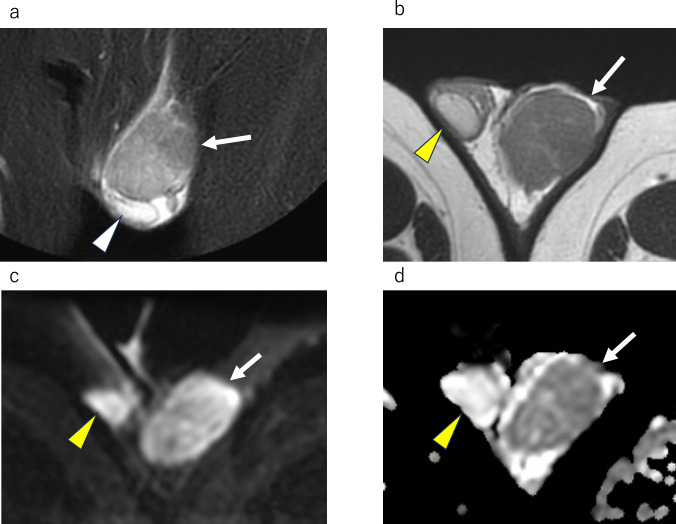
Leiomyosarcoma in a 60-year-old male with a left scrotal mass enlargement 3 months ago. **a** Coronal fat-suppressed T2-weighted MR image shows mild hypointense mass (white arrow) superior to the left testis (white arrowhead). There is no invasion of the testis. **b** Axial T2-weighted MR image shows mild hypointense mass with lobulated shape (white arrow). The yellow arrowhead indicates the normal right testis. **c** Axial diffusion weighted MR image shows a hyperintense mass (white arrow) comparable to that of the normal right testis (yellow arrowhead). **d** Apparent diffusion coefficient (ADC) images show that the mass (white arrow) has a low ADC value (0.874 × 10^−3^mm^2^/sec) compared to the normal right testis (yellow arrowhead)

MRI is valuable for evaluating spermatic cord leiomyosarcomas and providing insights into the extent of tumor spread. However, the imaging features of these tumors are often nonspecific, making qualitative diagnosis challenging [3, 29]. Spermatic cord leiomyosarcomas typically manifest as solid masses on MRI and exhibit heterogeneous enhancement within the scrotal region of the spermatic cord (Fig. 13).

The primary treatment for spermatic cord leiomyosarcoma is transinguinal radical orchiectomy, which involves complete surgical removal of the affected testis and surrounding structures, to achieve local control. Adjuvant radiation therapy may be recommended to reduce the risk of local recurrence, particularly in cases of high-grade lesions or tumors with aggressive features [29, 30]. Chemotherapy may also be considered in cases of high-grade lesions or in those with evidence of distal metastases with the aim of targeting systemic diseases and improving overall outcomes.

### Metastasis to the spermatic cord

Metastasis to the spermatic cord is rare, with an incidence ranging from 0.02 to 2.5% [31]. Metastasis to the spermatic cord most commonly originates from primary tumors in the stomach (60%), followed by the rectum, pancreas, and kidneys [32]. Interestingly, in some cases, metastases to the spermatic cord may be identified before detection of the primary tumor. Spermatic cord metastases may be associated with pain; however, they may also be asymptomatic.

Metastasis to the spermatic cord can occur through various pathways. The most common route is the retrograde lymphatic spread, followed by dissemination through adjacent structures [33]. Hematogenous metastasis, in which cancer cells travel through the bloodstream, has also been observed. Metastases may also occur via retrograde venous or vascular spread, although these routes are infrequent [33]. Among these five metastatic pathways, dissemination occurs more commonly in cases where the peritoneal sac is patent and is often accompanied by an inguinal hernia [33].

**Fig. 14:**
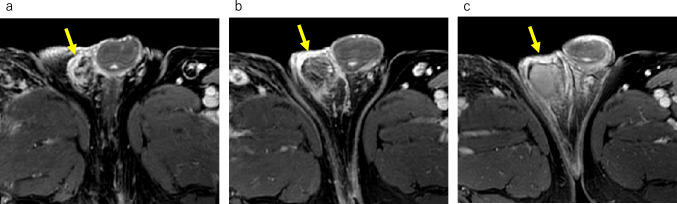
Metastasis to the spermatic cord in a 69-year-old male with right scrotal pain. Post rectal cancer surgery was performed 1 year ago. The histology of the primary tumor was signet-ring cell carcinoma. **a**–**c** Axial contrast-enhanced fat-suppressed T1-weighted MR image shows heterogeneously enhanced-thickening of the layer comprising the cremasteric muscle sandwiched between the internal and external spermatic fasciae (yellow arrows). Owing to the lack of a radical cure and severe pain, radical high-inguinal orchiectomy was performed, and the pathology showed solitary infiltrating tumor cells in the fatty and connective tissues of the spermatic cord

Spermatic cord metastases are typically diagnosed using CT or MRI. Diagnosis is relatively easy when there is a nodule or tumor in the inguinal canal accompanied by other metastases, such as in the lungs. However, in some cases, they may present as infiltrative lesions with indistinct borders, making them recognizable only as thickening fasciae without forming distinct masses (Fig. 14). In such cases, diagnosis can be challenging.

The treatment for metastases to the spermatic cord usually involves a multidisciplinary approach that includes surgical resection, chemotherapy, and/or radiation therapy [32]. The choice of treatment depends on factors such as the primary tumor site, extent of metastases, and overall health of the patient.

## Conclusion

MRI for scrotal masses is useful for assessing the extent and nature of the mass. Especially in extratesticular masses, benign lesions (fibrous pseudotumor, polyorchidism, adenomatoid tumors, and scrotal leiomyomas) present characteristic findings on MRI. Familiarity with these MRI findings will lead to accurate diagnosis and appropriate treatment.

## Data Availability

Data sharing is not applicable to this article as no new data were created or analyzed in this review.
